# Aggression in online gaming: the role of online disinhibition, social dominance orientation, moral disengagement and gender traits among Chinese university students

**DOI:** 10.3389/fpubh.2024.1459696

**Published:** 2024-11-06

**Authors:** Wenxuan Gan, Zichao Chen, Zhusheng Wu, Xia Huang, Fang Wang

**Affiliations:** ^1^School of Physical Education, Sichuan University, Chengdu, China; ^2^School of Tourism, Sichuan University, Chengdu, China

**Keywords:** online games, cyberaggressive behavior, gender traits, moral disengagement, online disinhibition

## Abstract

**Introduction:**

Aggressive behaviors in the online gaming world are frequent and have far-reaching negative effects.

**Method:**

To explore the factors and mechanisms of aggressive in games, we surveyed 945 university students using a framework of social dominance orientation, online disinhibition, moral disengagement, and aggression in gaming, and examined the moderating role of gender traits.

**Results:**

We found no direct relationship between online disinhibition and aggression in gaming; however, through the mediating role of moral disengagement, online disinhibition indirectly affected aggression in gaming behavior and enhanced social dominance orientation. Social dominance orientation predicted both moral disengagement and aggression in gaming behaviors, and the mediating effect of moral disengagement was confirmed through the indirect influence of moral disengagement on aggression in gaming behavior. Moreover, the moral disengagement mechanism significantly predicted aggression in gaming behavior. Furthermore, femininity and androgyny moderated both social dominance orientation toward moral disengagement and aggression in gaming, while masculinity and androgyny moderated the path from online disinhibition to social dominance orientation. Regarding the path from moral disengagement to aggression, all gender trait moderations were significant.

**Discussion:**

This study reveals the role of the moral disengagement mechanism in the process of game-related aggression, providing theoretical support for the explanation of aggressive behavior, which applies to players of any gender. Moreover, this study confirms the moderating role of gender. Unlike biological sex, gender traits are malleable; androgynous traits offer greater adaptability in various environments. Thus, prevention and intervention efforts against online aggression should include strengthening moral education and properly guiding and fostering androgynous gender traits.

## 1 Introduction

With the advancement of information technology, online gaming has emerged as a popular leisure activity (1). In 2024, the online gaming population reached approximately 332 million global, with China being the largest market, accounting for approximately 74.219 million online gamers (2). Considering this vast base, it is particularly crucial to study the social interactions among gamers and the effects thereof. Although social interaction in gaming is common and offers numerous benefits like social support (3), stress reduction (4), and health improvement (5), it also attracts online aggression and harassment (6). In the 18–24 years age group, 70% of gamers reported experiencing aggression, with 62% considering it a major issue (7). Online gaming is a major source of aggressive behaviors (8).

Aggressive behaviors in gaming environments can severely affect adolescents’ mental and physical development, leading to reduced self-esteem, increased truancy, a decreased sense of self-worth, despair, and mental disorders (9, 10). Compared to traditional aggressive behaviors, online aggression is more closely associated with victims’ suicidal tendencies (11, 12). The negative effects of aggressive behaviors are not limited to the online environment, as they affect all aspects of life and can last for a lifetime (13). Additionally, aggressive behaviors directly lead to player attrition and hinder the addition of new players, threatening the revenue of gaming companies (14, 15). The long-standing prevalence of aggression in games has caused significant ‘disasters’ for players and developers alike because the causes of aggression are complex, multifaceted, and difficult to eradicate.

The causes of Aggressive behaviors in gaming have become a significant topic of research in academia and various industries (16). Some studies have shown that as games incorporate more graphic violence and increasingly realistic scenarios, immersive and scenario-based gaming experiences promote the development of aggressive cognitions (17). Moreover, imbalances among players regarding level, skill, and external social capital contribute to aggressive behaviors. While these studies identify potential influencing factors, they less frequently examine the mechanisms behind aggression. This could partly be because the influencing factors in the aforementioned studies are public and apparent; however, in the anonymous, unregulated online environment, many aggressive behaviors are highly concealed. In such cases, victims may feel hurt but struggle to identify the aggression, making it difficult for victims to respond effectively, and for the players and gaming companies to recognize the aggressive behaviors. Therefore, this study introduces the concept of ‘moral disengagement’ (MD), exploring the mechanisms behind aggressive behavior in the weakly regulated spaces created by online anonymity. Moral disengagement is a cognitive mechanism through which an individual convinces themselves that behaviors violating personal moral standards are acceptable, enabling the execution of unethical and outrageous actions (18). This concept has been extensively applied to traditional aggressive behaviors (19), and can be considered to have good transferability to explain aggressive behaviors in the context of eSports gaming.

Furthermore, with the changing image of online gaming as being ‘for everyone’ (20), studies indicate that women are often seen as outsiders and may become targets of aggression and harassment as members of out-groups (21). However, there is controversy regarding the relationship between male and female university students and their experiences of cyber aggression. In a study of 695 undergraduate students, Walker found no statistically significant difference between men and women in experiencing aggressive behavior (22). In contrast, another study involving 666 undergraduate students from the Faculty of Education at Selçuk University, Turkey, found that as a form of aggressive behavior, cyberattack was more common among male students than female students (23). Using biological sex to predict bullying behavior has limited effectiveness. This may be explained by the notion that aggressive behavior is a form of personal socialization in which gender identification plays a significant role; the process of acquiring and developing gender traits is an individual’s identification with the gender role behaviors expected from societal culture and customs (24). Studies have found that gender role identification has a greater influence on aggressive behavior than biological sex (25). Although gender traits are closely related to aggressive behavior, few studies have explored the moderating role of gender traits in the context of online gaming. Therefore, this study incorporates gender trait variables into the model.

Given that online gaming is already a significant part of many university students’ digital lives, most existing research on cyberaggressive behavior has focused on middle and high school students, with relatively few studies targeting university students (26). In China, middle and high school students face greater academic pressure and study loads compared to university students. Upon entering university, students are no longer under the direct supervision of parents and teachers, which allows them to choose how to spend their time independently. This sudden freedom may lead many students to immerse themselves excessively in online gaming. According to statistics, as of June 2023, China had 1.079 billion internet users, with 13.9% aged 10–19 and 14.5% aged 20–29. The number of online gamers reached 550 million, accounting for 51.0% of the total internet population (27). It is rare for any other country globally to have such a large base, underscoring the importance of studying university students’ behaviors in online gaming. Moreover, Chinese society emphasizes collectivism. However, the anonymity in online games allows individuals to break free from collective norms present in real life, enabling players to exhibit more antisocial behaviors without being bound by societal rules (28). Based on this context, this study focused on Chinese university students and examined aggression in gaming as the dependent variable, exploring the underlying psychological mechanisms through the concept of moral disengagement. The study also incorporates online disinhibition, reflecting characteristics like anonymity and reduced self-restraint in online environments, as well as social dominance orientation, aligning with the competitive nature of gaming spaces and indicating social capital factors such as game levels and skills. Additionally, since students at this stage exhibit high personality plasticity, the study further investigates the moderating effect of gender roles on the relationships between these variables. From the perspective of the individual, this study aims to provide suggestions for the prevention and intervention of cyberattacks.

## 2 Literature review

### 2.1 Online disinhibition

Online disinhibition (OD) is defined as a decrease in self-restraint within the online world, accompanied by less concern for the consequences of one’s actions (29). Suler (30) described the online disinhibition effect as originating from six factors: dissociative anonymity, invisibility, asynchronicity, solipsistic introjection, dissociative imagination, and minimization of authority. The interaction of these factors complicates and amplifies the online disinhibition effect, creating an illusion of escaping punishment. As individuals in online gaming cannot see others, they worry less about the reactions to their actions, leading to increased boldness in engaging in atypical online behaviors (31). Research has indicated that OD is significantly associated with the occurrence of aggression (32). Lee (33) identified OD as the most potent predictor of involvement in aggression among African American students in a study concerning their use of the Internet and smartphones. Similarly, as part of the online world, aggression in online gaming has attracted scholarly attention. Research has shown that the competitiveness and violent content of video games can escalate player aggression (34, 35); yet, few studies have directly examined the link between OD and aggressive behaviors within gaming settings. Given the unique and complex nature of the online environment, this study includes OD in its model. Suler (30) described dissociative anonymity as a factor in which individuals, able to dissociate their online identities from their real-life ones, exhibit reduced self-restraint and expose their thoughts and actions more readily, which can provoke dominant and inequitable behaviors.

### 2.2 Social dominance orientation

Derived from social dominance theory, social dominance orientation (SDO) is defined as the degree to which individuals support a hierarchical system among social groups and the extent to which some groups dominate others (36). Groups at the top of society are dominant, while those at the bottom are subordinate; consequently, dominant social groups often engage in aggressive behaviors against subordinate groups (37). In the gaming world, gaming levels and skills are considered representations of social hierarchy and status. Higher levels signify a player’s success and capabilities in the game, depicting them as successful individuals (38). Aggressive social dominance theory suggests that groups with higher ranks or status endorse aggressive behaviors, as the lack of real-world constraints and the imbalance of power intensify online (39), leading to unrestrained dominant behaviors. OD not only activates potential SDO but also deepens its intensity. Studies on SDO and aggressive behaviors suggest that it can provides perpetrators with benefits, such as power, dominance, and popularity (40). Many youths involved in aggression state that they do so to elevate their social dominance status (41). Gaining dominance through aggression leads to social capital, which can then be used to coerce others or secure tangible rewards. These results illustrate the relationship between individuals with high SDO and aggressive behaviors to some extent. However, research indicates that SDO exhibits relative stability across different contexts and over time (42). Further, other studies have suggested that SDO is susceptible to socialization and prolonged exposure to specific social environments (43). Therefore, there is a need to explore new variables to enhance the explanatory power of the prediction models; this resulted in the introduction of MD as a mediating variable in this study. This aids in understanding the relationships among SDO, OD, and aggressive in gaming. The following hypotheses are posited in this study:

*Hypothesis 1*: Online disinhibition has a positive impact on aggression in gaming.

*Hypothesis 2*: Online disinhibition positively affects social dominance orientation.

*Hypothesis 3*: Social dominance orientation positively influences aggression in gaming.

### 2.3 Moral disengagement as a mediating variable

Bandura et al. (44) described MD as a cognitive process in which individuals rationalize their actions without feeling remorse, guilt, or self-censure despite knowing that their actions are morally wrong; it justifies destructive behaviors that violate their internal moral standards. Utilizing the mechanism of MD may facilitate aggressive behaviors in groups with high SDO. Research shows that SDO can indirectly influence the enactment of hate speech through high levels of MD (45). Low and Espelage (46) suggested that SDO or power is a primary motive for aggression in various contexts. However, most theories of aggression do not specifically address the potential impact of video games. Accordingly, we consider SDO as a potential influencing factor in aggressive behavior in online games.

Since Bandura’s initial empirical studies on adolescents, the role of MD in aggressive behavior has become a central research topic (47). Tanrikulu and Campbell (48) noted a distinct relationship between traditional aggression and MD; studies comparing MD levels between bullies and victims showed significantly higher levels in bullies. Most mechanisms of aggression can be traced back to aspects of MD. In traditional social settings, extensive research has been conducted on the correlation between MD and aggression (49, 50). However, with modernization, it is crucial to examine how online environments contribute to MD, thereby facilitating the emergence of aggression.

Online environments structurally support MD, potentially enhancing the use of specific disengagement mechanisms, and fostering cyber-aggressive behaviors. The absence of social cues in the online world may leave cyber aggressors with insufficient social information to accurately assess harm, complicating self-regulation and self-monitoring of their actions. They may employ the MD mechanism, ‘distortion of consequences,’ to project the interpretation of their actions onto victims, thus minimizing or distorting the consequences of their actions to reduce guilt (51). Pornari and Wood (49) suggested that the anonymity of the medium and the perceived distance from the victim may result in cyberbullies experiencing reduced feelings of guilt, shame, or empathy. This closely aligns with Bandura’s dehumanization mechanism within MD, where if there is a spatial or temporal disconnect between the act of harm and the resulting harm and the perpetrator cannot see the harm, it becomes easier to inflict harm on others. Furthermore, using information and communication technologies inherently removes emotional content from exchanges, resulting in structural dehumanization. Many studies have investigated MD in cyberaggressive behavior. A meta-analysis by Gini et al. (50) showed significant associations between MD and offline and cyberaggressive behavior, a finding echoed by Orue and Calvete (52). However, this hypothesis has not yet been widely validated; another study indicated that the link between MD and cyberaggressive behavior is insignificant (53).

Thus, the relationship between cyberaggressive behavior and MD remains disputed, with most scholars in previous studies generally focusing on the relationship between cyberaggressive behavior and MD, without considering its specific relevance in the context of online gaming. Video games have evolved into realistic and intricate worlds in which thousands of players interact, cooperate, and compete to achieve their gaming objectives (38). However, the gaming environment also serves as a venue for players to express their emotions freely. Most players view video games as fictional settings in which they can engage in activities that are impossible in real life, such as fighting or killing (54). Williams (55) suggested that aggressive games may have a normative effect on some players, potentially triggering MD mechanisms in such environments. Shafer (56) observed that some players make immoral choices in games, believing that their actions have no real consequences because ‘it’s just a game’, suggesting that MD mechanisms apply (51). Therefore, we posit the following hypotheses:

*Hypothesis 4*: Social dominance orientation has a positive impact on moral disengagement.

*Hypothesis 5*: Online disinhibition has a positive impact on moral disengagement.

*Hypothesis 6*: Moral disengagement has a positive impact on aggression in gaming.

### 2.4 Aggression in gaming

Cyberaggressive behavior, also known as cyberattack or online aggressive behavior, refers to aggressive behavior conducted via a broad range of information and communication technologies, such as social networking sites, email, chat programs, and text messages (49). Aggression in gaming (AIG) is a subset of cyberaggression and online gaming environments present several unique factors that make them particularly conducive to cyberaggression compared to general online spaces.

Firstly, games offer a more immersive experience. The extremity and realism of violent content immerse players in the virtual world, making them desensitized to aggressive behaviors. Players adopt the martial moral standards within the game as they embody their in-game characters (35, 57). Secondly, the competitive structure and ranking systems in many online games further exacerbate aggressive behavior. The empowerment granted by game levels digitizes player power, creating amplified differences between players. As quantified levels within the game space become symbols of player status and power, players must compete for rank or level, which can lead to heightened aggression, especially when players perceive their status as threatened. Higher-ranked players may engage in aggressive behaviors to maintain or enhance their ranking.

Additionally, personal attacks on opponents are common in games (58). Even in cooperative games, players may be harassed for making mistakes or failing to contribute to the team. Research has shown that antisocial behaviors in online games, such as griefing (unacceptable or antisocial behavior within the gaming context) and video game toxicity (59, 60), are commonplace in many gaming communities. These behaviors are often considered inherent parts of gaming culture. Consequently, the normalization of such behaviors has facilitated their spread, further fostering a unique culture of cyberaggression within gaming environments.

### 2.5 Gender traits as a moderating variable

Gender significantly influences human behavior, and Barlett and Coyne (61) proposed using it as a moderating factor to negate the effects of gender differences. Gender continues to be a focal point in aggression research, although conclusive findings are lacking (62, 63). Moyano et al. (64) discovered that men are more likely to perpetrate cyberaggressive behavior than women. However, other studies have indicated no statistically significant differences between boys and girls in the prevalence of engaging in or being victims of cyberaggressive behavior (65). Additionally, research has demonstrated a stronger correlation among boys than girls regarding overall MD, SDO, and involvement in physical aggression and cyberaggression (66). Menesini et al. (67) asserted that the relationship between MD and aggressive behavior is consistent across boys and girls. The ongoing debate suggests that the gender composition of studies does not significantly moderate cyberaggressive behavior. Therefore, there is a need for new moderating variables. Wright (58) provided a potential explanation for these differences, suggesting that masculine traits may be more significant than gender, noting that boys and girls with masculine traits are most likely to engage in cyberaggressive behavior. In current cyberaggressive behavior research, most studies address the connection with gender but overlook the link with gender temperament.

Terman and Miles (68) developed masculinity and femininity as measurable psychological constructs and laid the theoretical groundwork for understanding these traits. Masculinity and femininity describe the typical characteristics of males and females. Khan and Townsend (69) found that the traits of masculinity include a sense of superiority, dominance, authority, power, and success, whereas those of femininity involve warmth, sensitivity, nurturance, and interdependence (70, 71). Australian sociologist Connell (72) posited that, in most communities, men are violent toward women, seeing them as weaker and inferior. Society often views women as ‘naturally’ disadvantaged, always in need of male protection. Consequently, to adhere to social norms, both men and women unconsciously uphold their gender roles, with men learning to be aggressive and dominant, and women using indirect or more covert strategies of attack for self-protection (73).

Video games have traditionally been seen as a masculine space, and most developers and players are men. The tasks within games are often based on competition and aggression, embodying traditional masculine traits (74). In an anonymous gaming space, players often disregard their identities and conform to the dominant masculinized social identity of the space, which fosters hostility and aggression toward others, particularly outsiders. In such environments, Bandura’s MD mechanism of diffusion of responsibility is also triggered, where attacking ‘outsiders’ is excused by the notion that ‘everyone is doing it,’ thus minimizing feelings of guilt and blame. Additionally, players report that lower-ranked and female players often face hostility from male players. For some players, in competitive social environments such as gaming, their identity and status may feel threatened, exacerbating their SDO (75). Individuals with high SDO tend to exhibit more pronounced aggressive behaviors.

**Figure 1:**
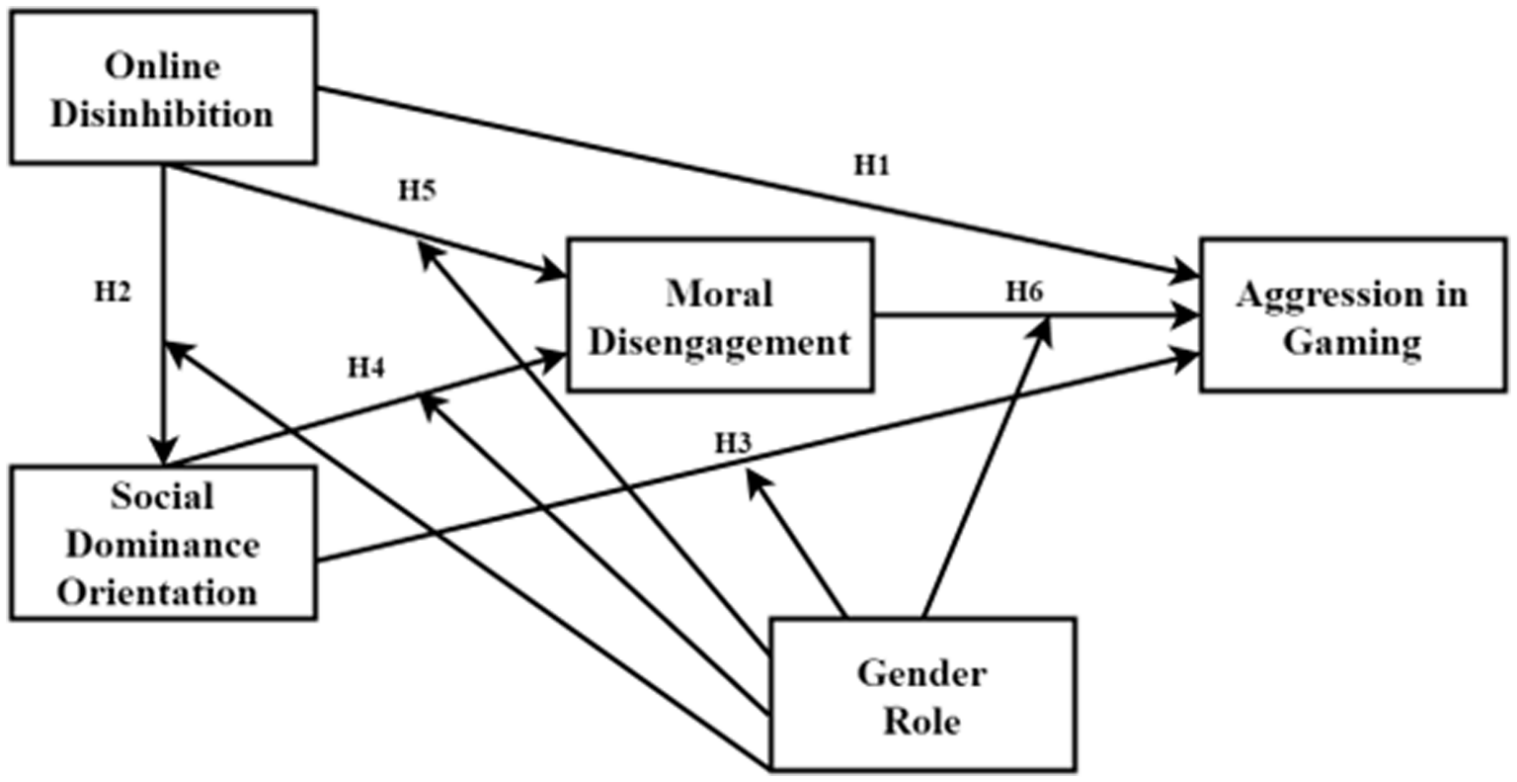
Theoretical framework.

Therefore, Figure 1 depicts the study framework regarding the moderating effect of gender roles on the relationships between SDO–MD, SDO–AIG, OD–SDO, OD–MD, and MD–AIG.

## 3 Method

### 3.1 Participants and procedures

An online survey was conducted among university students in China. Most participants were recruited offline from universities within Sichuan Province, China. Six graduate students, trained in survey techniques and with extensive experience, collected data from six universities within Sichuan. After informing the students of the survey’s purpose and obtaining their consent, they completed the questionnaires. The remaining questionnaires were distributed online through Wenjuanxing, the most widely used survey platform in China. The surveys were promoted via social media and WeChat groups to reach a broader audience. In the first part of the survey, we informed participants that their responses would be used solely for research purposes and kept confidential. During and after data collection, no information could identify individual participants.

In the absence of an adequate sampling frame and in line with previous research (76), a convenience sampling method was used to select participants. To minimize sample bias, the team collected data from multiple universities within Sichuan Province. As noted by Farrokhi and Mahmoudi-Hamidabad (77), convenience sampling can still enhance the reliability and validity of results by setting reasonable criteria and standards. This method is widely used in social science research. Survey distribution and data collection took place over 2 months in April 2024. Prior to the official data collection, a pilot study was conducted in March 2023 at three universities in Sichuan Province, where 200 questionnaires were distributed and 183 valid responses were obtained. The data from the pilot study were used to test the reliability and validity of the scales, leading to the elimination of items that did not meet the standards and adjustments to the wording of certain items based on respondents’ feedback.

A large-scale survey was subsequently conducted, distributing a total of 1,100 questionnaires, with 945 valid responses obtained, resulting in an 85.9% response rate. We collected basic demographic information from respondents, including gender, age, and education level. We also included metrics related to game engagement, such as game duration, frequency, and social interactions. As shown in Table 1, the respondent demographics included a balanced gender distribution, with 42% male and 58% female participants. In terms of age, 80.4% of respondents were aged 17–22, and 12.8% were aged 23–25. Regarding education level, 87.9% of respondents held a bachelor’s degree, and 12.1% held a degree above the undergraduate level. Regarding game engagement, 14% had been playing games for more than 10 years, while 44.7% had been playing for 1–5 years. In terms of weekly gaming frequency, 15.4% played more than 10 times a week, and 41.5% played 4–10 times a week. For session length, 42.4% of respondents played games for 1–3 h on average per session, and 6.6% played for more than 6 h. In terms of social interactions in games, 61.4% had fewer than five close friends with whom they frequently interacted in the gaming environment.

**Table 1:** Demographic profile of respondents.

Demographics	Frequency (*N* = 945)	%
Gender		
Male	397	42
Female	548	58
Age		
17–22	760	80.4
23–25	121	12.8
26–30	64	6.8
Academic degree		
Bachelor	831	87.9
Master	67	7.1
Doctor	47	5.0
Gaming engagement		
How long have you been playing games since your first encounter?		
0–6 months	137	14.5
7–12 months	108	11.4
1–3 years	178	18.8
3–5 years	245	25.9
5–10 years	145	15.3
Over 10 year	132	14.0
How often do you log in to play games per week?		
Less than once	167	17.7
1–3 times	240	25.4
4–6 times	257	27.2
7–10 times	135	14.3
More than 10 times	146	15.4
On average, how long do you play games each session?		
Less than 1 h	346	36.6
1–3 h	401	42.4
4–6 h	136	14.4
Over 6 h	62	6.6
How many friends do you frequently contact within the game?		
Fewer than 5 friends	580	61.4
5–10 friends	228	24.1
10–20 friends	39	4.1
20–40 friends	53	5.6
More than 40 friends	45	4.8

### 3.2 Instruments

The survey is divided into three sections: the first involves basic sociodemographic characteristics. The second includes a refined gender role scale adapted from Zhang et al. (78), featuring 14 and 12 items for masculine and feminine traits, respectively. The items are rated on a 7-point scale, ranging from 1 ‘completely disagree’ to 7 ‘completely agree,’ with higher scores signifying greater alignment with masculine or feminine traits. The scores for each participant on the femininity and masculinity scales were calculated and compared with the respective medians; the participants were then classified into masculine, feminine, and androgynous categories.

The third section includes an aggression in gaming questionnaire developed for this study, consisting of four dimensions. Originally in English, this questionnaire was translated and back-translated to ensure accuracy in translation and expression (79). First, a native Chinese-speaking professional translator converted the original scale into Chinese. This was followed by a panel of three bilingual scholars who back-translated the version into English to ensure accuracy and equivalence. All items related to the theoretical constructs in this study were measured using a 7-point Likert scale ranging from 1 ‘strongly disagree’ to 7 ‘strongly agree’.

Social dominance orientation was assessed using items adapted from (80, 81). The scale measures individuals’ preference for hierarchy and dominance within social groups. Example items include: “It might be a good thing that some people are at the top and others are at the bottom.” Online disinhibition was measured using items based on Suler (30). This scale assesses individuals’ tendency to express themselves more freely online than in face-to-face interactions. Example items include: “It is easier to connect with others through CTs than talking in person.”

Moral disengagement was measured using items adapted from (82, 83). This scale evaluates the extent to which individuals disengage from moral standards to justify unethical behavior. Example items include: “Special tactics to win a game are justifiable.” Aggression in gaming was measured using items from (84, 85). This scale assesses aggressive behaviors directed toward others within the gaming environment. Example items include: “I have verbally harassed others openly in games.”

### 3.3 Data analysis

This study conducted exploratory factor analysis using SPSS 26.0. The internal consistency of each construct was assessed using Cronbach’s *α*. A Cronbach’s α value greater than 0.70 indicates acceptable reliability, suggesting that the items within each scale consistently measure the same construct. We assessed both convergent validity and discriminant validity to ensure the appropriateness of the measurement model, convergent validity was evaluated to ensure that items within each construct were highly correlated and effectively measured the same underlying construct. Discriminant validity was assessed to ensure that each construct was unique and distinct from others in the model. Additionally, we performed the KMO (Kaiser–Meyer–Olkin) test to measure sampling adequacy and Bartlett’s test of sphericity to confirm that factor analysis was appropriate.

Following the recommendations of Hair et al. (86), principal component analysis with Varimax rotation was applied, and items with factor loadings below 0.60 were removed. Thus, item SDO4 from the social dominance orientation scale, MD6 from moral disengagement, and AIG7 from aggression in gaming were deleted; finally, there were a total of 20 effective items. In this study, the initial eigenvalues accounted for 32.838% of the variance, and the cumulative squared loadings after rotation were 71.061%. Thus, this study does not have a common method bias issue (87).

To ensure the data met the assumptions for parametric analyses, we examined the distribution of all variables. The skewness values ranged from −0.664 to 1.676, and the kurtosis values ranged from −1.323 to 1.980, both of which were within the acceptable range of less than ±2 (88). Therefore, the data were considered approximately normal, justifying the use of Maximum Likelihood (ML) estimation for further analysis. We employed AMOS 24.0 for SEM to test the relationships between variables and assess the research hypotheses.ML estimation was used to estimate model parameters. Goodness-of-fit indices, including Chi-square (*χ*^2^), Comparative Fit Index (CFI), Tucker–Lewis Index (TLI), and Root Mean Square Error of Approximation (RMSEA), were reported to evaluate how well the hypothesized model fits the observed data. The mediation effect of Moral Disengagement was tested using the bootstrapping method with 5,000 resamples. To examine the moderation effects, we used multi-group analysis in AMOS. Specifically, the sample was divided into subgroups based on gender traits, and we tested whether the path coefficients between key constructs varied significantly across these subgroups.

## 4 Results

### 4.1 Reliability and validity test

The results showed that the Cronbach’s alpha for the masculinity scale of the gender role questionnaire was 0.915 and for the femininity scale was 0.906. The factor loadings for each dimension of the aggression in gaming questionnaire ranged from 0.705 to 0.908. The KMO value was 0.903. Reliability tests showed that Cronbach’s alpha for all scales ranged from 0.855 to 0.925, composite reliability ranged from 0.840 to 0.916, and average variance extracted (AVE) of all scales ranged from 0.573 to 0.776 (Tables 2, 3). According to Fornell and Larcker (89), the factor reliability should be greater than 0.70, and AVE should be greater than 0.50. Thus, all dimensions of the aggression in gaming questionnaire met these criteria. The square root of the AVE values (bolded in the table) is greater than the correlation coefficients among the dimensions listed below the diagonal. There are significant differences in validity among the dimensions (Table 3). Therefore, the questionnaire met the desired reliability and convergent validity criteria.

**Table 2:** Discriminatory validity.

Construct research	Item	Unstd	S.E.	*z*-value	*P*	std.	CR	AVE	SMC
SDO	It might be a good thing that some people are at the top and others are at the bottom.	1				0.871	0.912	0.776	0.759
	If some people were content with their place, we would have fewer problems.	1.027	0.028	36.409	***	0.904			0.817
	Sometimes, certain groups must be kept in their place.	1.028	0.030	34.582	***	0.867			0.752
OD	It is easier to connect with others through CTs than talking in person.	1.217	0.056	21.579	***	0.776	0.894	0.629	0.602
	The Internet is anonymous so it is easier for me to express my true feelings or thoughts.	1.269	0.055	23.150	***	0.842			0.709
	It is easier to write things online that would be hard to say in real life because you do not see the other’s face.	1.224	0.054	22.562	***	0.817			0.667
	It is easier to communicate online because you can reply anytime you like.	1.222	0.053	22.895	***	0.831			0.691
	I have an image of the other person in my head when I read their e-mail or messages online.	1				0.688			0.473
MD	Special tactics to win a game are justifiable.	1				0.701	0.840	0.573	0.491
	Relative to actual illegal activities, bullying in games is trivial.	1.063	0.046	22.864	***	0.838			0.702
	Mocking others in a game is insignificant since it does not result in actual harm.	1.068	0.046	23.154	***	0.855			0.731
	If the game administrators fail to act against bullying, blaming players for bullying should not be considered justified.	0.922	0.054	17.081	***	0.606			0.367
AIG	I have verbally harassed others openly in games.	1				0.785	0.916	0.648	0.616
	I have insulted opponents in games due to their skill levels.	1.178	0.041	28.682	***	0.844			0.712
	I have mocked others for their lower rank or level in games.	1.025	0.038	27.148	***	0.808			0.653
	I have tormented others in games.	1.217	0.041	29.375	***	0.860			0.740
	I have used specific game tactics to humiliate others.	1.214	0.043	28.459	***	0.839			0.704
	I have intentionally underperformed or played carelessly in games.	0.885	0.040	21.923		0.679			0.461

SDO, social dominance orientation; OD, online disinhibition; MD, moral disengagement; AIG, aggression in gaming. ***p < 0.001.

**Table 3:** Convergent and discriminant validity results.

	SDO	MD	OD	AIG
SDO	0.881			
MD	0.169	0.757		
OD	0.278	−0.083	0.793	
AIG	0.187	0.655	−0.040	0.805

SDO, social dominance orientation; OD, online disinhibition; MD, moral disengagement; AIG, aggression in gaming.

**Figure 2:**
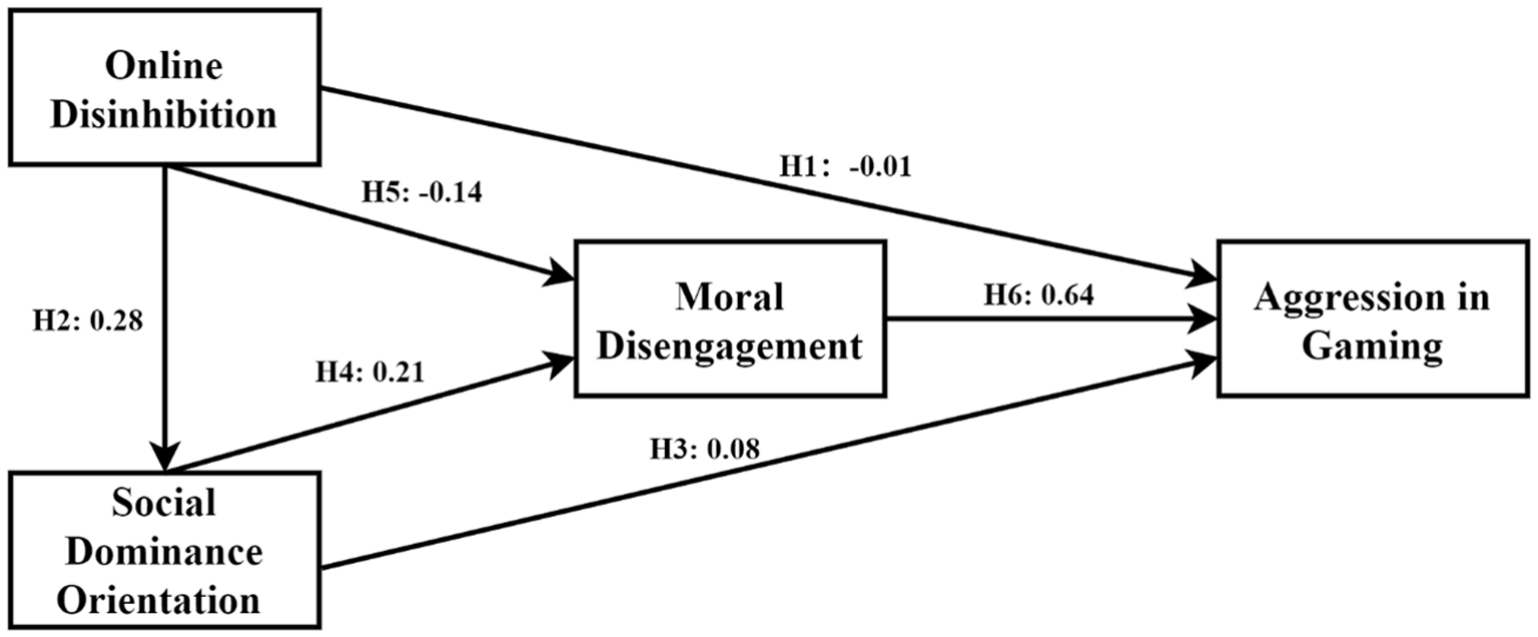
Structural relationship framework.

Based on maximum likelihood estimates, commonly used fit indices were utilized to assess the model’s fit, as shown in Table 4 and Figure 2. Following the recommendations of Hair et al. (86) and others, and drawing from the experience of other researchers, a GFI, NFI, and CFI greater than 0.90 indicate a good fit (90). Although there is no absolute standard for RMR, values less than 0.05 suggest a close model fit (91). RMSEA values below 0.05 indicate a close fit, while values between 0.05 and 0.08 suggest an acceptable fit (92). The SRMR value should be ≤0.08 (93). After necessary assessments of measurement models and model fit, this study conducted a structural model analysis. The analysis paths were assessed using 5,000 bootstrap samples and a 95% confidence interval. According to Zhao et al. (94), an indirect effect [independent variable → mediator → dependent variable] with a 95% CI that does not include zero indicates a significant mediation effect. Overall, these findings indicate that our model is relatively satisfactory.

**Table 4:** Index table of SEM fitness.

Model fitting degree	Standard	Actual fitting degree of model	Reach standard
ML *χ*^2^	As small as possible	419.545	Reach standard
df	As small as possible	129	Reach standard
Normed Chi-sqr (*χ*^2^/df)	1 < *χ*^2^/df < 5	3.252	Reach standard
SRMR	<0.08	0.030	Reach standard
GFI	>0.9	0.950	Reach standard
AGFI	>0.9	0.933	Reach standard
RMSEA	<0.08	0.049	Reach standard

The results indicate that six hypotheses were tested in the conceptual model, five of which were supported (see Table 5): H1: Online Disinhibition (OD) does not significantly affect Aggression in Gaming (AIG) [*β* = −0.009, *p* = 0.758 > 0.001]; H2: OD has a positive impact on social dominance orientation (SDO) [*β* = 0.278, *p* < 0.001]; H3: SDO positively influences AIG [*β* = 0.082, *p* < 0.01]; H4: SDO has a positive effect on moral disengagement (MD) [*β* = 0.208, *p* < 0.001]; H5: OD negatively impacts MD [*β* = −0.141, *p* < 0.001]; and H6: MD positively affects AIG [*β* = 0.640, *p* < 0.001].

**Table 5:** Outcomes of SEM analysis (*N* = 945).

Hypothesis	Path			Path coefficient (β)	S.E.	C.R.	*P*	Supported?
H1	OD	→	AIG	−0.009	0.031	−0.308	0.758	No
H2	OD	→	SDO	0.278	0.054	7.642	***	Yes
H3	SDO	→	AIG	0.082	0.021	2.630	0.009**	Yes
H4	SDO	→	MD	0.208	0.026	5.407	***	Yes
H5	OD	→	MD	−0.141	0.039	−3.649	***	Yes
H6	MD	→	AIG	0.640	0.041	15.753	***	Yes

***p < 0.001, **p < 0.01. SDO, social dominance orientation; OD, online disinhibition; MD, moral disengagement; AIG, aggression in gaming.

### 4.2 Mediation and moderation effects

This study used the Bootstrap method to test for mediating effects, as listed in Table 6. Hypothesis 1: the direct effect of OD on AIG was not significant [*β* = −0.009, *p* = 0.758 > 0.001]; however, OD had an indirect effect on AIG through MD, amounting to −0.092 [SE = 0.027, |*Z*| = |−3.407| > 1.96, *p* = 0.001 < 0.01], with the 95% confidence interval not including zero, indicating a full mediation effect. Hypothesis 3: the direct effect of SDO on AIG was significant [*β* = 0.082, *p* < 0.001], and SDO had an indirect effect on AIG through MD, amounting to 0.091 [SE = 0.015, *Z* = 6.067 > 1.96, *p* = 0.000 < 0.01], with the 95% confidence interval not including zero, indicating a partial mediation effect.

**Table 6:** Intermediary effect test.

Relationships	Point estimate	Product of coefficient		Bootstrap 5,000 times 95% CI				
				Bias-corrected		Percentile		
		S.E.	*Z*	Lower	Upper	Lower	Upper	*p*
Indirect effects								
OD-MD-AIG	−0.092	0.027	−3.407	−0.148	−0.040	−0.147	−0.040	0.001**
SDO-MD-AIG	0.091	0.015	6.067	0.062	0.122	0.061	0.122	0***
Total IE	−0.001	0.026	−0.038	−0.054	0.048	−0.055	0.048	0.959
Direct Effect	0.056	0.022	2.545	0.014	0.102	0.013	0.102	0.008**
Total Effect	0.055	0.036	1.153	−0.016	0.124	−0.016	0.124	0.127

*p < 0.05, **p < 0.01, ***p < 0.001.

Table 7 lists the path coefficient estimates from the multi-group SEM model. In Hypothesis 2 [OD → SDO path], masculinity [*β* = 0.395, SE = 0.098, *p* < 0.05] and androgyny [*β* = 0.351, SE = 0.073, *p* < 0.05] are significant, whereas femininity [*β* = −0.095, SE = 0.097, *p* = 0.292 > 0.05] is not, indicating that masculinity and androgyny have moderating effects in the OD → SDO path, while femininity does not. In Hypothesis 3 [SDO → AIG path], femininity [*β* = 0.203, SE = 0.067, *p* = 0.016 < 0.05] and androgyny [*β* = 0.144, SE = 0.028, *p* = 0.014 < 0.05] are significant, whereas masculinity [*β* = 0.01, SE = 0.066, *p* = 0.91 > 0.01] is not, suggesting that femininity and androgyny have moderating effects in the SDO → AIG path, while masculinity does not. In Hypothesis 4 [SDO → MD path], femininity [*β* = 0.360, SE = 0.060, *p* < 0.01] and androgyny [*β* = 0.211, SE = 0.04, *p* = 0.002 < 0.01] are significant, whereas masculinity [*β* = 0.161, SE = 0.089, *p* = 0.14 > 0.01] is not, indicating that femininity and androgyny have moderating effects in the SDO → MD path, while masculinity does not.

**Table 7:** Estimation results of multi-groups path coefficients.

Path			Masculinity			Femininity		Androgyny	
			Estimate		S.E.	Estimate	S.E.	Estimate	S.E.
H2	OD	→	SDO	0.395***	0.098	−0.095(0.292)	0.097	0.351***	0.073
H3	SDO	→	AIG	0.01(0.91)	0.066	0.203(0.016*)	0.067	0.144(0.014*)	0.028
H4	SDO	→	MD	0.161(0.14)	0.089	0.360***	0.060	0.211(0.002**)	0.040
H5	OD	→	MD	−0.105(0.327)	0.091	−0.115(0.184)	0.062	−0.111(0.104)	0.048
H6	MD	→	AIG	0.587***	0.101	0.450***	0.110	0.584***	0.066

*p < 0.05, **p < 0.01, ***p < 0.001. SDO, social dominance orientation; OD, online disinhibition; MD, moral disengagement; AIG, aggression in gaming.

In Hypothesis 5 [OD → MD path], masculinity [*β* = −0.105, SE = 0.091, *p* = 0.327 > 0.05], femininity [*β* = −0.115, SE = 0.062, *p* = 0.184 > 0.05], and androgyny [*β* = −0.111, SE = 0.048, *p* = 0.104 > 0.05] are all not significant, indicating that there are no moderating effects in the OD → MD path. In Hypothesis 6 [MD → AIG path], masculinity [*β* = 0.587, SE = 0.101, *p* < 0.01], femininity [*β* = 0.450, SE = 0.110, *p* < 0.01], and androgyny [*β* = 0.584, SE = 0.066, *p* < 0.01] are all significant, indicating that moderating effects exist in the MD → AIG path.

## 5 Discussion and conclusions

### 5.1 Discussion

With online gaming increasingly becoming a common leisure activity, aggression in these games has become widespread, and garnered significant scholarly attention. This study explored the psychological mechanisms underlying cyberaggressive behavior during gaming. It examined the relationships and internal mechanisms between OD, MD, SDO, and gaming aggression through a mediating moderation model as well as the moderating effect of gender traits.

No significant relationship between OD and aggressive behaviors in gaming was found, which contradicts previous research (95) that suggested that disinhibition in online environments promotes verbal abuse, bullying, and other forms of cyberaggression (96). These contradictory findings prompted a re-evaluation of the applicability of the online disinhibition effect across different online settings. Gaming environments with clear rules and strict enforcement against aggression somewhat regulate player behavior. For example, Riot Games, the parent company of the popular game ‘League of Legends,’ discovered a positive correlation between game losses and toxic behavior. Consequently, they enhanced the reporting mechanisms within the user interface design and intensified penalties to reduce toxicity in the game. Additionally, the asynchrony described by Suler (30), where sending and receiving information does not have to occur simultaneously, may not apply in gaming, where players’ actions receive immediate feedback, possibly encouraging more cautious behavior. This study confirms the link between OD and MD, noting that an increase in OD could reduce MD, a finding that contradicts previous research (97). This may be related to reverse acting, a concept introduced by Freud that refers to the defense mechanisms used by individuals to handle unacceptable desires and conflicts, necessitating strengthened defenses to prevent these impulses from emerging. OD effects, such as anonymity, invisibility, and asynchrony, make individuals feel less restrained in online environments, leading to potentially unethical and aggressive behaviors (98). When players anticipate this, reverse acting may trigger internal vigilance that paradoxically constrains their behavior to prevent them from engaging in unethical actions, thereby reducing MD. This finding confirms that MD completely mediates the negative relationship between OD and aggression in gaming. When MD is reduced, individuals become more aware of the moral consequences of their actions and thus avoid cyberaggressive behaviors.

The results of this study confirm Hypothesis 2, showing that OD enhances SDO. Power and status differences are common features of cyberaggressive behaviors, even in online environments. Gender and rank influence the status within the game (99), leading to women and lower-ranked players often being viewed as outsiders in gaming environments. Anyone perceived as an outsider (based on demographic characteristics, skill levels, or other behaviors) may threaten the existence of gaming spaces characterized by exclusivity. Therefore, some players may infringe on these groups to defend their dominant status. Moreover, the highly competitive nature of gaming environments, in which players form teams with strangers in a relatively short time to compete against other teams, is a significant feature (100). Once the factors representing status are threatened, the SDO intensifies. In addition, the significant social identity prompted by the online disinhibition effect encourages the process of deindividuation. Players who cling to their perceived insider social identities are more exclusionary toward other groups, and higher levels of SDO may increase the likelihood of negative social behaviors in online environments due to this deindividuation process.

Hypothesis 4, which states that SDO is significantly related to MD, is confirmed in this study. This indicates that the higher a player’s SDO, the greater their level of MD. Social dominance orientation is used to measure the extent to which certain groups dominate others (101, 102). Moral disengagement is an active regulatory mechanism and players with a strong desire to maintain group dominance are more likely to trigger mechanisms thereof. This study also confirms the relationships among SDO, MD, and AIG, with SDO indirectly influencing aggressive behaviors through MD. This aligns with previous research showing that individuals, particularly adolescents with a strong inclination to uphold the group hierarchy, actively engage in MD to make aggressive statements (45). Bandura et al. (44) suggested that players who perceive themselves as having a high status may actively engage in MD to bully other players. The repetitive nature of aggressive behaviors in games normalizes justifications for unethical behavior, thus perpetuating frequent occurrences of aggression in gaming.

This study also confirms Hypothesis 3, establishing a positive correlation between SDO and aggressive behaviors. Espelage et al. (103) noted that aggression in online gaming contexts is related to the pursuit of status, with factors such as game rank, skill, and gender representing status and power.

Regarding Hypothesis 6, the operational mechanisms of MD under aggressive behaviors were validated. Players with higher levels of MD are more likely to engage in aggression in gaming through mechanisms such as moral justification, dehumanization, and the minimization of consequences. For instance, bullies may use trash talk, teasing, or joking to normalize their aggressive behaviors or justify that aggressive behaviors in the virtual world do not cause real harm to others; thereby, they employ MD mechanisms to defend their actions and reduce psychological stress, thus facilitating aggression.

This study also considers gender traits as a moderating variable. The results showed that the players’ gender traits had moderating effects on the SDO–MD, SDO–AIG, OD–SDO, and MD–AIG pathways. Femininity and androgyny had positive moderating effects on the SDO–MD and SDO–AIG paths, whereas masculinity was not significant. Individuals with high SDO and masculine traits may not need to rely on MD to alleviate internal conflicts when engaging in dominant behaviors, as their values and behaviors already justify such dominance (43). Furthermore, masculine individuals may view aggressive behaviors as a natural way to display power and maintain dominance, thereby directly exhibiting aggressive behaviors. In contrast, femininity emphasizes empathy, care, and cooperation; therefore, those with higher femineity may experience psychological struggles during unethical behaviors, which may force them to rely on MD mechanisms to alleviate their internal moral conflict.

Because feminine traits suppress aggressive behaviors, individuals with these traits may adopt more covert forms of aggression. Cyberaggressive behavior is described as a covert form of aggression, especially when it involves social exclusion and the maintenance of status hierarchies within peer groups (104). Individuals with feminine personality traits tend to endorse and support covert aggression, particularly among feminine girls and others who highly value feminine traits (105). Additionally, research indicates that since women are a primary target of online aggression, one of their strategies for coping with aggression in gaming involves adopting an aggressive personality. Therefore, feminine personality traits are related to SDO and AIG. Androgyny has been proven to be a gender role identity type that perfectly blends masculine and feminine traits, allowing one to actively modulate their behavior in various situations (106). Therefore, it is easier for androgynous individuals to reconcile conflicts among aggressive behavior, dominance tendencies, and behavioral traits through MD mechanisms.

Masculinity and androgyny significantly modulate the OD–SDO pathway, likely because individuals with masculine traits more readily exhibit dominance tendencies under high OD conditions, as OD amplifies their pursuit of power and control (107). It may manifest through verbal attacks, threats, or exerting pressure to control others. Androgynous individuals can flexibly adjust their behavior in online settings by utilizing the disinhibition effect to combine masculine dominance behaviors with feminine empathy, thus displaying dominance tendencies online more easily. They may exhibit toughness and authority in competitive online environments, while using feminine traits to actively offer help and be more attentive to others’ feelings in non-competitive contexts, balancing the guilt brought about by dominance behaviors. The moderating effect of femininity was not significant, possibly because it emphasizes traits that typically conflict with dominance tendencies, and are culturally expected to manifest gentleness and cooperation rather than dominance and control. These societal expectations might limit the likelihood of individuals with high OD and feminine traits exhibiting dominant behaviors in online environments.

For the MD–AIG pathway, all temperaments were significantly modulated, indicating that the impact of moral disengagement on aggression in gaming is ever-present in anonymous online environments. This highlights the fact that each gender role has the potential to engage in aggressive behavior through MD mechanisms, with every online gamer potentially becoming a bully in certain contexts. Similarly, for the OD–MD pathway, all temperaments were not significant, indicating that the MD mechanism does not vary with anonymity and its active regulatory mechanism is unaffected by various gender traits in online environments.

### 5.2 Theoretical contributions

This study makes several theoretical contributions to the understanding of aggression in gaming environments. First, by focusing on SDO as a critical variable, the research expands on existing studies that have typically focused on game level or skill as indicators of power dynamics in gaming. SDO offers a more comprehensive framework to examine hierarchical structures within gaming environments, highlighting how individuals with strong dominance orientations are more likely to engage in aggressive behaviors to maintain or enhance their perceived status. This theoretical expansion underscores the role of social power in aggressive behaviors and provides a novel lens through which to study online gaming aggression.

Second, the study sheds light on the mediating role of MD in gaming contexts. Previous research has identified MD as a mechanism through which individuals rationalize unethical behavior, but its specific role in online gaming environments has not been thoroughly explored. This study confirms that MD significantly mediates the relationship between SDO and aggression in gaming, offering a clearer understanding of how players justify harmful behaviors in competitive gaming settings. This finding demonstrating its applicability in virtual environments. For example, players may use moral disengagement mechanisms to justify their aggressive behavior in the virtual world, such as the excuse that attacking others in a game does not cause real harm to people in the real world, thus reducing the psychological pressure they feel.

Thirdly, this study validated the moderating role of gender roles, expanding the theoretical understanding of how gender role socialization influences online aggressive behavior. The results showed that feminine and androgynous roles significantly moderated the relationships between SDO, MD, and AIG, while masculine traits did not exhibit a significant effect. This finding differs from previous studies that demonstrated a significant correlation between masculine traits and aggressive behavior. Feminine roles, which emphasize empathy and cooperation, are more likely to experience internal moral conflict when faced with aggressive behavior, leading to the use of moral disengagement mechanisms to alleviate discomfort, thereby contributing to covert aggression in online environments. Androgynous roles, due to their flexibility in different contexts, demonstrated stronger adaptability in online environments.

Lastly, the study contributes to the understanding of OD and its relationship with moral disengagement and social dominance orientation. While prior research has established a link between OD and various forms of online aggression, this study found no direct relationship between OD and aggression in gaming. Suggests that the unique characteristics of gaming environments—such as real-time feedback and strict enforcement of rules—may moderate the typical disinhibition effects observed in other online contexts. This insight invites future research to explore the boundary conditions of OD across different types of online platforms.

### 5.3 Practical implications

This study has several practical implications. Game content should promote cooperation rather than competition. Hypothesis 3 SDO enhances AIG, and the study confirmed this relationship. Given that power and status differences are key features of aggressive in online gaming environments, game developers can take measures to minimize these dominance hierarchies. One approach is to create more balanced gaming environments where player status is less tied to aggressive behavior. For example, developers could implement non-competitive game modes that emphasize cooperation over competition or reduce the visible rankings of players to lessen the focus on dominance. Developers might also create in-game incentives for prosocial behavior, such as rewards for teamwork or collaborative achievements, to counteract the aggressive behaviors driven by dominance.

Correctly guiding and cultivating androgynous gender roles. Unlike biological sex, gender roles are malleable, and androgynous traits demonstrate stronger adaptability to various environments. Healthy and positive gender characteristics can be promoted through media and public opinion. Schools should foster masculine traits such as bravery, strength, and decisiveness in girls and feminine traits such as attentiveness and empathy in boys. Educational philosophies should move away from traditional gender role models to properly consider gender differences and foster healthy and equal gender perceptions.

### 5.4 Limitations and future research

Although this study has made contributions, several limitations need to be considered. First, the sample was limited to university students, which restricts the generalizability of the findings. Future research should include a broader population to test whether the observed relationships apply to different contexts. The focus on Chinese university students also limits the cross-cultural applicability of the results. Second, the cross-sectional design of the study limits the ability to infer causal relationships between variables. While the use of Structural Equation Modeling (SEM) provided insights into the relationships between SDO, MD, and AIG, longitudinal studies are needed to explore how these relationships evolve over time. Finally, this study did not directly explore the impact of game mechanics (such as ranking systems or team dynamics) on aggressive behavior. Future research could incorporate these variables to gain a more detailed understanding of how specific game features promote or mitigate aggression in games.

Based on these limitations, future research should adopt a longitudinal design, which would help to understand more dynamically how these factors interact and influence behavior over time. Additionally, investigating the bystander effect in gaming environments is a key next step. Bystanders often make up the largest group in online games, and their passive or active participation can significantly influence the occurrence of aggressive behavior. Understanding how bystanders contribute to or inhibit aggressive behavior can lead to more effective interventions to reduce cyber aggression. Finally, the role of game mechanics (such as ranking systems, reward structures, and team dynamics) should be further explored. These game-specific features may exacerbate or alleviate aggression, and understanding their impact could provide valuable insights for game developers to create safer and more inclusive gaming environments.

## Data Availability

The original contributions presented in the study are included in the article/supplementary material, further inquiries can be directed to the corresponding author.
